# Abdominal Adiposity and Total Body Fat as Predictors of Cardiometabolic Health in Children and Adolescents With Obesity

**DOI:** 10.3389/fendo.2020.00579

**Published:** 2020-09-04

**Authors:** Binghan Jin, Hu Lin, Jinna Yuan, Guanping Dong, Ke Huang, Wei Wu, Xuefeng Chen, Li Zhang, Jinling Wang, Xinyi Liang, Yangli Dai, Xiaoqin Xu, Xuelian Zhou, Mingqiang Zhu, Guohua Li, Wayne S. Cutfield, Paul L. Hofman, José G. B. Derraik, Junfen Fu

**Affiliations:** ^1^Department of Endocrinology, The Children's Hospital, Zhejiang University School of Medicine, National Clinical Research Center for Child Health, Hangzhou, China; ^2^Liggins Institute, University of Auckland, Auckland, New Zealand; ^3^A Better Start – National Science Challenge, University of Auckland, Auckland, New Zealand; ^4^Department of Women's and Children's Health, Uppsala University, Uppsala, Sweden

**Keywords:** android-to-gynoid-fat ratio, blood pressure, body composition, glucose, insulin sensitivity, lipids, metabolism, NAFLD

## Abstract

**Objective:** We aimed to assess the role of adipose tissue distribution in cardiometabolic risk (in particular insulin sensitivity) in a population of children and adolescents with obesity.

**Methods:** In this cross-sectional study, participants were 479 children and adolescents with obesity (322 boys and 157 girls) aged 3 to 18 years attending the Children's Hospital at Zhejiang University School of Medicine (Hangzhou, China). Clinical assessments included anthropometry, body composition (DXA scans), carotid artery ultrasounds, and OGTT. Insulin sensitivity was assessed using the Matsuda index. Participants were stratified into groups by sex and pubertal stage. Key predictors were DXA-derived android-to-gynoid-fat ratio (A/G) and total body fat percentage (TBF%).

**Results:** Irrespective of sex and pubertal stage, there was a strong association between increasing A/G (i.e., greater abdominal adiposity) and lower insulin sensitivity. In multivariable models, every 0.1 increase in A/G was associated with a reduction in insulin sensitivity in prepubertal boys [−29% (95% CI −36%, −20%); *p* < 0.0001], pubertal boys [−13% (95% CI −21%, −6%); *p* = 0.001], and pubertal girls [−16% (95% CI −24%, −6%); *p* = 0.002]. In contrast, TBF% was not associated with insulin sensitivity when A/G was adjusted for, irrespective of pubertal stage or sex. In addition, every 0.1 increase in A/G was associated with increased likelihood of dyslipidemia in prepubertal boys [adjusted odds ratio (aOR) 1.62 (95% CI 1.05, 2.49)], impaired glucose tolerance in pubertal boys [aOR 1.64 (95% CI 1.07, 2.51)] and pubertal girls [aOR 1.81 (95% CI 1.10, 2.98)], and odds of NAFLD in both prepubertal [aOR 2.57 (95% CI 1.56, 4.21)] and pubertal [aOR 1.69 (95% CI 1.18, 2.40)] boys. In contrast, higher TBF% was only associated with higher fasting insulin and ALT in pubertal boys, being also predictive of NAFLD in this group [aOR 1.15 per percentage point (95% CI 1.06, 1.26)], but was not associated with the likelihood of other cardiometabolic outcomes assessed in any group.

**Conclusions:** A/G is a much stronger independent predictor of cardiometabolic risk factors in children and adolescents with obesity in China, particularly glucose metabolism.

## Introduction

Children and adolescent obesity has reached epidemic proportions in many countries, posing a serious public health challenge (1). Worldwide, the number of children and adolescents aged 5 to 19 years with obesity has risen 10-fold since 1975 (2). It is estimated that 41 million children under the age of 5 years and over 340 million youth aged 5–19 years had overweight or obesity in 2016 (2). Notably, there has been an accelerated rise in the prevalence of childhood and adolescent obesity in both low- and middle-income countries, especially in Asia (2, 3). In China, the prevalence of overweight and obesity has been steadily rising since the early 1990s, particularly among boys and youth living in urban areas (4). For example, in large coastal cities between 1985 and 2000, the prevalence of obesity increased from 0.3 to 9.1% among boys and from 0.2 to 4.8% among girls aged 7–18 years (4). Thus, by 2013, the prevalence of obesity among Chinese children and adolescents was 6.9% for boys and 2.8% for girls (5).

Obesity is associated with many adverse effects on both physical and psychological health across the life span, from childhood to adulthood (1). In particular, obesity is associated with increased risk of high blood pressure, dyslipidemia, non-alcoholic fatty liver disease (NAFLD), impaired glucose tolerance, insulin resistance, type 2 diabetes, and the metabolic syndrome in childhood (6–9). Importantly, childhood obesity tracks into adulthood (10), and is associated with an increased risk of cardiovascular morbidity and mortality in adulthood (11–13), independent of adult body mass index (BMI) (12).

Obesity is characterized by excessive weight gain, particularly through an excessive accumulation of fat in adipocytes (14). Consequently, there is a chronic state of inflammation, insulin resistance, and other adverse cardiometabolic outcomes (14). Notably, a seminal study by Vague in 1956 showed that obesity-related adverse health consequences were mostly associated with an android pattern of obesity or upper body fat accumulation (15). There is now considerable evidence that cardiometabolic complications from adult obesity are closely related to body fat distribution, with a gender dimorphism (16, 17). In men, adipose tissue tends to accumulate in the upper body (both subcutaneous and visceral), leading to a pattern of android fat distribution characterized by a convex belly and an apple-shaped body (18). Conversely, in women, adipose tissue accumulates mostly subcutaneously, especially thighs and hips, leading to a pear-shaped body (i.e., a pattern of gynoid fat distribution) (18). These sexually dimorphic patterns of body fat deposition tend to establish during adolescence and continue into adulthood (19).

Imaging techniques such as dual-energy X-ray absorptiometry (DXA) scans enable us to evaluate patterns of fat deposition, including levels of abdominal adiposity based on the android-to-gynoid-fat ratio (A/G). Several studies have shown an association between A/G and the risk of cardiometabolic disease in adults (20–22). However, a limited number of studies have focused on children and adolescents (23–27), with paucity of data on the influence of pubertal development. Further, the reported associations between cardiometabolic risk and total vs. abdominal adiposity is inconsistent. Some studies showed both total body fat and abdominal fat were similarly predictive of cardiometabolic risk factors (28, 29), whereas, others have proposed abdominal fat is more predictive (23, 27). Thus, we aimed to assess the role of adipose tissue distribution in cardiometabolic risk in a population of youth with obesity, in particular the relative contributions of total body fat percentage (TBF%) and A/G.

## Methods

### Study Participants

Participants in this study were children and adolescents who presented with obesity to the Department of Endocrinology at the Children's Hospital at Zhejiang University School of Medicine (Hangzhou, China), between January 2016 and April 2018. All participants were voluntarily brought to the hospital by their parents due to concerns about their children's excessive weight gain. After presentation to the hospital's outpatient endocrinology clinic, these patients were admitted to the Department of Endocrinology where they underwent a range of clinical assessments. A follow-up outpatient appointment was arranged, so that appropriate medical referral and/or treatment was provided if any abnormalities were identified. All participants were free of medication known to affect energy metabolism and none had pre-diagnosed hereditary diseases or evidence of overt chronic heart, lung, or kidney disease, or other chronic illness.

### Ethics Approval

This study was approved by the Medical Ethics Committee of the Children's Hospital of Zhejiang University School of Medicine. Written informed consent was obtained from parents or guardians, as well as from the children (where appropriate, otherwise verbal assent was sought).

### Clinical Assessments

All participants underwent clinical assessments at the Department of Endocrinology. Most assessments were performed by endocrinologists, otherwise they were done by specialized nurses at this clinic. During the assessment, caregivers were asked when they had first noticed that their children had a weight issue; this self-reported measure was recorded as the approximate duration of obesity.

### Anthropometry

Participants were weighed on electronic scales (regularly calibrated) while barefoot and wearing only light clothing, with weight recorded to the nearest 0.1 kg. Standing height was measured to the nearest millimeter using a Harpenden stadiometer, also barefoot, and while maintaining as straight posture. Body mass index (BMI) was subsequently calculated. Height, weight, and BMI were then transformed into standard deviation scores (SDS) adjusted for age and sex as per WHO standards (30), with obesity defined as a BMI SDS ≥1.645 (i.e., at or above the 95^th^ percentile). Waist circumference was measured with non-stretchable tape to the nearest mm on bare skin after a gentle exhalation, at the horizontal level of the midpoint between the lowest rib and the iliac crest.

### Pubertal Staging

Sexual maturity was assessed by pediatric endocrinologists and defined according to Tanner stage (31). Pre-pubertal status corresponded to Tanner stage 1, and pubertal as stages 2 to 5 (31).

### Body Composition

Body composition was measured by DXA scans using a Hologic Discovery Wi scanner (Hologic Inc, Bedford, MA, USA). Key parameters of interest were TBF% and A/G. The latter parameter was derived from the estimates of both android fat and gynoid fat automatically calculated by the manufacturer's software, based on standardized partitioning of the body as described by Bazzochi et al. (32) and Shepherd et al. (33), so that the adiposity estimates are largely independent of age and ethnicity.

### Insulin Sensitivity

All patients underwent an oral glucose tolerance test (OGTT) after an overnight fast, with blood samples drawn at 0, 30, 60, 90, and 120 min for glucose and insulin. The glucose load was administered at 1.75 g per kg of body weight, to a maximum of 75 g. Insulin sensitivity was assessed using the Matsuda index (34), calculated as:

$$\begin{array}{l} Matsuda\;index=\frac{10,000}{\sqrt{\left(\text{fasting plasma glucose x fasting plasma insulin}\right)\text{ x }\left(\text{mean OGTT glucose x mean OGTT insulin}\right)}} \end{array}$$

The Matsuda index is strongly correlated with the hyperinsulinemic euglycaemic clamp (35), and also has high reproducibility during multiple measures (36). Glycated hemoglobin (HbA1c) was also measured.

### Additional Blood Markers

Additional venous blood samples at the start of the OGTT were taken to measure lipid profile and liver function. Parameters measured included triglycerides, low-density lipoprotein cholesterol (LDL), high-density lipoprotein cholesterol (HDL), total cholesterol, aspartate transaminase (AST), and alanine transaminase (ALT). In addition, uric acid was also measured as a marker of inflammation (37).

### Carotid Intima-Media Thickness

The intima-media thickness of the left carotid artery (CIMT) was measured using a Doppler ultrasound (38), probe 11L, LOGIQ E9 (General Electric, USA). Measurements were made with the patient lying down on a supine position, with the neck fully exposed, and the head slightly bent to the right. Intima-media thickness was measured three consecutive times at ~1 cm proximal to the carotid bulb, and it was defined as the mean distance between the leading edge of the lumen-intima interface and the leading edge of the media-adventitia interface of the posterior wall (39).

### Blood Pressure

Systolic (SBP) and diastolic (DBP) blood pressures were measured using a sphygmomanometer on the right upper arm while seated, and after a 5-min rest. Two measurements were taken and the average value used.

### Adverse Outcomes

The list of adverse cardiometabolic outcomes is provided in Table 1, alongside their respective diagnostic criteria.

**Table 1:** Diagnostic criteria for adverse outcomes as adopted in our study.

**Condition**	**Age**	**Minimum diagnostic criteria**	**References**
Impaired fasting glucose	All	fasting plasma glucose ≥5.6 and <7.0 mmol/L	(40)
Impaired glucose tolerance	All	a) 2-h plasma glucose concentration ≥7.8 and <11.1 mmol/L^#^; AND b) Fasting plasma glucose concentration <7.0 mmol/L	(40)
Type 2 diabetes mellitus	All	a) 2-h plasma glucose concentration ≥11.1 mmol/L^#^; OR b) Fasting plasma glucose ≥7.0 mmol/L	(40)
Abnormal glycaemia	All	a) Impaired fasting glucose; OR b) Impaired glucose tolerance; OR c) Type 2 diabetes mellitus	(40)
Hypertension	<10 years	a) Systolic blood pressure ≥ 120 mmHg; OR b) Diastolic blood pressure ≥80 mmHg	(41)
	≥10 years	a) Systolic blood pressure ≥ 130 mmHg; OR b) Diastolic blood pressure ≥85 mmHg	(41)
Dyslipidaemia	All	a) Triglycerides ≥1.7 mmol/l; OR b) HDL <1.03 mmol/l	(42)
Metabolic syndrome	≥10 years	a) Obesity; AND any two of: b) Abnormal glycaemia [as defined above]; c) Hypertension [as defined above]; d) Dyslipidaemia [as defined above]	(42)
Hyperuricaemia	All	Plasma uric acid ≥5.5 mg/dl	(43)
NAFLD	All	(i) Hepatic imaging results are compatible with fatty liver; and (ii) Secondary causes have been ruled out; with or without metabolic risk factors	(44)

*NAFLD, non-alcoholic fatty liver disease*.

# *Parameter measured after a 75-g glucose load from an oral glucose tolerance test*.

### Statistical Analyses

Our study population was stratified into four groups according to sex and pubertal status: pre-pubertal boys, pre-pubertal girls, pubertal boys, and pubertal girls. The distribution of all outcome parameters was examined, and where appropriate they were log-transformed to approximate a normal distribution. Simple linear associations between A/G and TBF% and cardiometabolic parameters were evaluated using Pearson's correlation coefficients.

Potential collinearity between A/G and TBF% within subgroups were also examined using Pearson's correlation coefficients, with high collinearity defined as |r|≥0.5 (45). As there were no cases of high collinearity (Supplementary Table 1), multiple linear regressions were run including both A/G and TBF% as independent variables within each subgroup. Results are provided as β coefficients and respective 95% confidence intervals (CI), as well as standardized coefficients. These models also included as covariates testicular volume for pubertal boys and age for pubertal girls, except for models examining blood pressure outcomes for which the respective covariates were replaced with height.

Generalized linear regression models were also run to assess the likelihood of adverse metabolic outcomes, adopting the same covariate adjustments as described above. These results are provided as adjusted odds ratio (aOR) and respective 95% CI.

Analyses were performed in SPSS v24 (IBM Corp, Armonk, NY, USA) and SAS v9.4 (SAS Institute, Cary, USA). All tests were two-tailed, with statistical significance set at *p* < 0.05 and without adjustment for multiple comparisons.

## Results

Our study population consisted of 479 children and adolescents with obesity with a mean age of 10.7 years, who had a parent-reported median duration of obesity of ~5 years. Participants were 139 pre-pubertal boys, 17 pre-pubertal girls, 183 pubertal boys, and 140 pubertal girls (Table 2; Supplementary Figure 1). In light of the limited number of pre-pubertal girls in our study population, these were excluded from further analyses. The distributions of A/G and TBF% in the three examined groups are shown in Supplementary Figure 2.

**Table 2:** Demographic and clinical characteristics of our study population from Hangzhou (China).

		**Study population**	**Pre-pubertal boys**	**Pre-pubertal girls**	**Pubertal boys**	**Pubertal girls**
***n***		479	139	17	183	140
Demography	Age (years)	11.0 [9.3, 12.4]	9.8 [8.5, 11.1]	7.9 [6.6, 9.4]	12.0 [10.9, 12.9]	11.2 [9.7, 12.8]
Clinical history	Obesity duration (years)	5.0 [3.0, 8.0]	5.0 [3.0, 8.0]	5.0 [2.0, 6.0]	5.0 [3.2, 8.0]	6.0 [3.0, 9.0]
Anthropometry	Height SDS	1.12 (1.02, 1.22)	1.16 (0.99, 1.34)	1.66 (1.11, 2.20)	1.16 (1.00, 1.32)	0.96 (0.78, 1.13)
	BMI SDS	3.07 (3.01, 3.14)	3.47 (3.34, 3.59)	3.45 (2.91, 4.00)	2.99 (2.90, 3.07)	2.75 (2.66, 2.85)
	Waist circumference (cm)	89.9 (88.9, 91.0)	88.9 (87.2, 90.7)	77.9 (72.3, 83.6)	94.6 (93.1, 96.1)	86.4 (84.2, 88.5)
Development	Precocious puberty	31 (6.5%)	n/a	n/a	5 (2.7%)	26 (18.6%)
Glucose metabolism	Matsuda index	2.14 (2.02, 2.26)	2.48 (2.23, 2.75)	2.78 (2.00, 3.86)	1.93 (1.78, 2.10)	2.05 (1.83, 2.29)
	Fasting glucose (mmol/l)	5.38 (5.34, 5.42)	5.33 (5.27, 5.40)	5.21 (4.97, 5.44)	5.40 (5.34, 5.45)	5.43 (5.35, 5.51)
	Fasting insulin (μIU/ml)	22.9 (20.8, 23.1)	18.5 (16.8, 20.2)	18.5 (12.2, 24.7)	23.7 (21.8, 25.5)	23.5 (21.1, 25.9)
	HbA1c (mmol/mol)	40.3 (39.7, 40.9)	41.1 (40.0, 42.2)	39.8 (36.0, 43.7)	40.2 (39.3, 41.2)	40.3 (39.7, 40.9)
Blood pressure	Systolic (mmHg)	124 (123, 126)	123 (120, 125)	120 (112, 127)	126 (124, 128)	124 (122, 126)
	Diastolic (mmHg)	72 (71, 73)	71 (69, 73)	69 (63, 75)	72 (71, 73)	72 (70, 74)
Liver function	ALT (U/l)	31.6 (29.6, 33.9)	33.5 (29.3, 38.3)	23.2 (17.6, 30.5)	34.2 (30.7, 38.1)	28.1 (24.8, 31.8)
	AST (U/l)	29.3 (28.1, 30.7)	30.5 (27.9, 33.5)	25.8 (21.9, 30.5)	29.8 (27.8, 32.0)	28.0 (25.9, 30.4)
Lipid profile	Total cholesterol (mmol/l)	4.33 (4.24, 4.41)	4.45 (4.30, 4.60)	4.33 (3.94, 4.71)	4.29 (4.15, 4.43)	4.25 (4.10, 4.40)
	Triglycerides (mmol/l)	1.22 (1.18, 1.27)	1.17 (1.09, 1.27)	1.02 (0.82, 1.28)	1.26 (1.18, 1.34)	1.26 (1.17, 1.35)
	LDL (mmol/l)	2.74 (2.68, 2.80)	2.83 (2.72, 2.94)	2.80 (2.50, 3.10)	2.70 (2.61, 2.80)	2.68 (2.58, 2.79)
	HDL (mmol/l)	1.27 (1.25, 1.29)	1.30 (1.26, 1.34)	1.31 (1.16, 1.46)	1.26 (1.22, 1.30)	1.25 (1.21, 1.29)
Inflammatory marker	Uric acid (μmol/l)	398 (390, 406)	376 (362, 389)	358 (326, 390)	427 (412, 442)	388 (373, 402)
Atherosclerosis marker	CIMT (mm)	0.57 (0.56, 0.58)	0.57 (0.55, 0.59)	0.60 (0.54, 0.66)	0.58 (0.56, 0.59)	0.55 (0.53, 0.57)

*Age and obesity duration data are medians [quartile 1, quartile 3]; precocious puberty data are n (%); all other data are means (95% confidence intervals)*.

*ALT, alanine transaminase; AST, aspartate transaminase; BMI SDS, body mass index standard deviation score; CIMT, carotid intima-media thickness; HbA1c, glycated hemoglobin; HDL, high-density lipoprotein cholesterol; LDL, low-density lipoprotein cholesterol; n/a, not applicable; SDS, standard deviation score*.

### Pre-pubertal Boys

Among the younger boys, there was a strong association between increasing A/G and greater insulin resistance, in stark contrast to TBF% (Table 3). Specifically, increasing A/G was correlated with lower insulin sensitivity assessed by the Matsuda index (*r* = −0.44; *p* < 0.0001; Supplementary Figure 3), higher fasting insulin (*r* = 0.38; *p* < 0.0001), and greater HbA1c (*r* = 0.17; *p* = 0.043) levels (Table 3). Increasing A/G was also correlated with higher ALT (*r* = 0.24; *p* = 0.004) and uric acid (*r* = 0.29; *p* = 0.001) levels (Table 3).

**Table 3:** Linear associations between the android-to-gynoid-fat ratio (A/G), total body fat percentage (TBF%), and cardiometabolic parameters among pre-pubertal boys with obesity in Hangzhou (China).

**Pre-pubertal boys**			**Correlation (pearson's)**				**Multiple regression**					
			**A/G**		**TBF%**		**A/G**^**‡**^			**TBF%**		
			***r***	***p***	***r***	***p***	β (95% CI)^*^	**Std β**	***p***	β (95% CI)^*^	**Std β**	***p***
Glucose metabolism	Matsuda index^†^	136	**−0.44**	**<0.0001**	−0.11	0.20	**−0.34 (−0.45**, **−0.22)**	**−0.45**	**<0.0001**	−0.03 (−0.06, 0.01)	−0.11	0.16
	Fasting glucose (mmol/l)	139	0.00	0.99	0.07	0.42	0.00 (−0.08, 0.09)	0.001	0.99	0.01 (−0.02, 0.04)	0.07	0.42
	Fasting insulin (μIU/ml)	139	**0.38**	**<0.0001**	0.13	0.13	**4.68 (2.79, 6.57)**	**0.38**	**<0.0001**	0.52 (−0.08, 1.12)	0.14	0.09
	HbA1c (mmol/mol)	139	**0.17**	**0.043**	0.04	0.61	**1.34 (0.05, 2.63)**	**0.17**	**0.043**	0.11 (−0.30, 0.52)	0.05	0.59
Blood pressure	Systolic (mmHg)	139	0.08	0.33	0.08	0.36	1.37 (−1.39, 4.13)	0.08	0.33	0.41 (−0.46, 1.29)	0.08	0.35
	Diastolic (mmHg)	139	0.05	0.60	−0.02	0.87	0.49 (−1.39, 2.37)	0.05	0.60	−0.05 (−0.65, 0.55)	−0.01	0.87
Liver function	ALT (U/l)^†^	139	**0.24**	**0.004**	0.01	0.90	**0.23 (0.05, 0.39)**	**0.24**	**0.004**	0.00 (−0.05, 0.06)	0.01	0.88
	AST (U/l)^†^	139	0.10	0.25	0.06	0.49	0.07 (−0.05, 0.18)	0.10	0.25	0.01 (−0.02, 0.05)	0.06	0.48
Lipid profile	Total cholesterol (mmol/l)	139	0.16	0.06	0.05	0.57	0.17 (−0.01, 0.35)	0.16	0.06	0.02 (−0.04, 0.08)	0.05	0.55
	Triglycerides (mmol/l)^†^	139	0.14	0.09	0.02	0.79	0.08 (−0.01, 0.18)	0.14	0.09	0.00 (−0.03, 0.04)	0.02	0.78
	LDL (mmol/l)	137	0.11	0.20	0.03	0.69	0.09 (−0.05, 0.22)	0.11	0.20	0.01 (−0.03, 0.052)	0.04	0.68
	HDL (mmol/l)	137	−0.14	0.11	−0.08	0.37	−0.04 (−0.09, 0.01)	−0.14	0.11	−0.01 (−0.02, 0.01)	−0.08	0.35
Inflammatory marker	Uric acid (μmol/l)	139	**0.29**	**0.001**	**−0.18**	**0.035**	**29.8 (13.0, 44.6)**	**0.29**	**<0.0001**	**5.7 (0.7, 10.7)**	**0.18**	**0.026**
Atherosclerosis marker	CIMT (mm)	131	−0.10	0.24	−0.02	0.87	−0.013 (−0.034, 0.009)	−0.11	0.24	0.001 (−0.007, 0.006)	−0.02	0.85

*Data are Pearson's correlation coefficients (r) and respective p-values; and β coefficients (95% confidence intervals), standardized coefficients (Std β), and respective p-values. Associations that are statistically significant (p <0.05) are shown in bold*.

*ALT, alanine transaminase; AST, aspartate transaminase; CI, confidence interval; CIMT, carotid intima-media thickness; HbA1c, glycated hemoglobin; HDL, high-density lipoprotein cholesterol; LDL, low-density lipoprotein cholesterol*.

* *Models included both the A/G and TBF% as independent variables*.

† *These parameters were log-transformed to approximate a normal distribution, in which case the percentage change in the outcome response was obtained using the formula exp(β) (Supplementary Methods)*.

‡ *β coefficients are shown in association with a change of 0.1 in A/G*.

Multiple regression models confirmed these associations, with every 0.1 increase in A/G associated with a 29% reduction in insulin sensitivity (95% CI −36%, −20%; *p* < 0.0001) (Supplementary Methods). Further, a 0.1 increase in A/G was associated with increases of 4.7 μIU/ml in fasting insulin (*p* < 0.0001) and 1.3 mmol/mol in HbA1c (*p* = 0.043), as well as increases in both ALT and uric acid levels (Table 3). Conversely, TBF% was only associated with uric acid levels (*p* = 0.026), but the standardized coefficients showed that the contribution of A/G to variation in uric acid levels was considerably greater than that of TBF% (Std β 0.29 and 0.18, respectively; Table 3).

### Pubertal Boys

A nearly identical pattern to that observed amongst the pre-pubertal group was seen for pubertal boys (Table 4). An increase in A/G was correlated with lower insulin sensitivity (−0.26; *p* < 0.0001; Supplementary Figure 3) and higher fasting insulin (*r* = 0.16; *p* = 0.027), as well as increased ALT (*r* = 0.20; *p* = 0.007) and uric acid levels (*r* = 0.26; *p* < 0.0001). For TBF%, the only association was an inverse correlation with triglycerides levels (*r* = −0.22; *p* = 0.002; Table 4).

**Table 4:** Linear associations between the android-to-gynoid-fat ratio (A/G), total body fat percentage (TBF%), and cardiometabolic parameters among pubertal boys with obesity in Hangzhou (China).

**Pubertal boys**			**Correlation (pearson's)**				**Multiple regression**					
			**A/G**		**TBF%**		**A/G**^**‡**^			**TBF%**		
		***n***	***r***	***p***	***r***	***p***	β (95% CI)^*^	**Std β**	***p***	β (95% CI)^*^	**Std β**	***p***
Glucose metabolism	Matsuda index^†^	181	**−0.26**	**<0.0001**	0.02	0.78	**−0.14 (−0.23**, **−0.06)**	**−0.25**	**0.001**	−0.01 (−0.03, 0.01)	−0.08	0.35
	Fasting glucose (mmol/l)	182	0.05	0.49	−0.03	0.70	0.03 (−0.04, 0.09)	0.06	0.42	0.00 (−0.02, 0.01)	−0.01	0.93
	Fasting insulin (μIU/ml)	183	**0.16**	**0.027**	0.08	0.30	**2.11 (0.18, 4.03)**	**0.16**	**0.032**	**0.51 (0.06, 0.97)**	**0.18**	**0.028**
	HbA1c (mmol/mol)	182	0.09	0.23	−0.01	0.90	0.59 (−0.39, 1.57)	0.09	0.24	0.00 (−0.22, 0.22)	0.00	0.98
Blood pressure	Systolic (mmHg)	180	0.09	0.26	−0.05	0.52	−0.21 (−2.39, 1.96)	−0.02	0.85	0.19 (−0.30, 0.68)	0.06	0.44
	Diastolic (mmHg)	180	0.07	0.35	0.03	0.67	0.30 (−1.31, 1.91)	0.03	0.72	0.20 (−0.16, 0.57)	0.09	0.27
Liver function	ALT (U/l)^†^	183	**0.20**	**0.007**	0.14	0.06	**0.17 (0.05, 0.28)**	**0.22**	**0.004**	**0.03 (0.01, 0.06)**	**0.18**	**0.026**
	AST (U/l)^†^	183	0.10	0.17	0.13	0.09	0.06 (−0.01, 0.13)	0.12	0.11	0.01 (0.00, 0.03)	0.12	0.14
Lipid profile	Total cholesterol (mmol/l)	183	−0.01	0.85	−0.01	0.91	0.00 (−0.15, 0.15)	−0.001	0.99	−0.01 (−0.04, 0.03)	−0.03	0.69
	Triglycerides (mmol/l)^†^	183	−0.04	0.61	**−0.22**	**0.002**	−0.03 (−0.10–0.03)	−0.07	0.33	**−0.03 (−0.04**, **−0.01)**	**−0.26**	**0.002**
	LDL (mmol/l)	183	−0.02	0.78	0.06	0.45	−0.01 (−0.11, 0.09)	−0.02	0.85	0.01 (−0.01, 0.03)	0.07	0.43
	HDL (mmol/l)	183	−0.09	0.24	0.09	0.22	−0.01 (−0.05, 0.03)	−0.03	0.68	0.00 (−0.01, 0.01)	0.02	0.81
Inflammatory marker	Uric acid (μmol/l)	183	**0.26**	**<0.0001**	−0.13	0.09	**22.0 (7.5, 36.5)**	**0.21**	**0.003**	1.2 (−2.25, 4.65)	0.05	0.49
Atherosclerosis marker	CIMT (mm)	170	0.03	0.67	0.10	0.18	0.004 (−0.013, 0.021)	0.04	0.63	**0.004 (0.000, 0.008)**	**0.19**	**0.029**

*Data are Pearson's correlation coefficients (r) and respective p-values; and β coefficients (95% confidence intervals), standardized coefficients (Std β), and respective p-values. Associations that are statistically significant (p <0.05) are shown in bold*.

*ALT, alanine transaminase; AST, aspartate transaminase; CI, confidence interval; CIMT, carotid intima-media thickness; HbA1c, glycated hemoglobin; HDL, high-density lipoprotein cholesterol; LDL, low-density lipoprotein cholesterol*.

* *Models included the A/G, TBF%, and testicular volume as independent variables; where the outcome was blood pressure, models adjusted for height instead of testicular volume*.

† *These parameters were log-transformed to approximate a normal distribution, in which case the percentage change in the outcome response was obtained using the formula exp(β) (Supplementary Methods)*.

‡ *β coefficients are shown in association with a change of 0.1 in A/G*.

In the multiple regression models, every 0.1 increase in A/G was associated with a 13% reduction in insulin sensitivity (95% CI −21%, −6%; *p* = 0.001) among pubertal boys (Table 4). However, both A/G and TBF% were independently associated with higher fasting insulin levels (β = 2.1, *p* = 0.032; β = 0.51, *p* = 0.028; respectively), with the associations with each predictor being of a similar magnitude (Std β 0.16 and 0.18, respectively) (Table 4). Both A/G and TBF% similarly affected ALT levels (Table 4). Of note, every one percentage point increase in TBF% was associated with a 0.004 mm increase in CIMT (*p* = 0.029; Table 4).

### Pubertal Girls

In this group, TBF% was not correlated with any of the cardiometabolic outcomes assessed (Table 5). In contrast, A/G was once again associated with markers of glucose homeostasis, liver function, and inflammation (Table 5). Increasing A/G was correlated with lower insulin sensitivity (*r* = −0.41; *p* < 0.0001; Supplementary Figure 3), but higher fasting insulin (*r* = 0.37; *p* < 0.0001), ALT (*r* = 0.46; *p* < 0.0001), AST (*r* = 0.33; *p* < 0.0001), and uric acid levels (*r* = 0.37; *p* < 0.0001). In addition, A/G was positively correlated with total cholesterol (*r* = 0.24; *p* = 0.004) and LDL (*r* = 0.26; *p* = 0.002) levels, as well as CIMT (*r* = 0.19; *p* = 0.036; Table 5).

**Table 5:** Linear associations between the android-to-gynoid-fat ratio (A/G), total body fat percentage (TBF%), and cardiometabolic parameters among pubertal girls with obesity in Hangzhou (China).

**Pubertal Girls**			**Correlation (pearson's)**				**Multivariable regression**					
			**A/G**		**TBF%**		**A/G**^**‡**^			**TBF%**		
		**n**	***r***	***p***	***r***	***p***	**β (95% CI)**	**Std β**	***p***	**β (95% CI)**	**Std β**	***p***
Glucose metabolism	Matsuda index^†^	134	**−0.41**	**<0.0001**	0.05	0.60	**−0.17 (−0.28**, **−0.06)**	**−0.30**	**0.002**	0.01 (−0.02, 0.04)	0.07	0.43
	Fasting glucose (mmol/l)	137	0.07	0.40	−0.04	0.64	0.06 (−0.03, 0.14)	0.14	0.21	−0.01 (−0.03, 0.01)	−0.09	0.34
	Fasting insulin (μIU/ml)	137	**0.37**	**<0.0001**	0.00	0.99	**3.44 (1.09, 5.79)**	**0.28**	**0.004**	−0.10 (−0.71, 0.52)	−0.03	0.76
	HbA1c (mmol/mol)	135	0.11	0.19	−0.02	0.85	0.70 (−0.06, 0.15)	0.13	0.16	−0.09 (−0.36, 0.19)	−0.06	0.53
Blood pressure	Systolic (mmHg)	137	0.20	0.019	−0.09	0.30	1.13 (−0.89, 3.15)	0.11	0.27	−0.24 (−0.78, 0.29)	−0.08	0.37
	Diastolic (mmHg)	137	0.21	0.013	−0.02	0.80	1.22 (−0.61, 3.06)	0.13	0.19	−0.06 (−0.55, 0.43)	−0.02	0.80
Liver function	ALT (U/l)^†^	140	**0.46**	**<0.0001**	−0.03	0.77	**0.27 (0.15, 0.38)**	**0.41**	**<0.0001**	−0.02 (−0.05, 0.01)	−0.12	0.17
	AST (U/l)^†^	140	**0.33**	**<0.0001**	0.01	0.96	**0.16 (0.07, 0.24)**	**0.38**	**<0.0001**	−0.01 (−0.03, 0.01)	−0.11	0.22
Lipid profile	Total cholesterol (mmol/l)	139	**0.24**	**0.004**	0.02	0.78	**0.20 (0.04, 0.37)**	**0.25**	**0.016**	−0.01 (−0.05, 0.03)	−0.05	0.59
	Triglycerides (mmol/l)^†^	139	0.16	0.06	−0.01	0.94	0.05 (−0.02, 0.13)	0.14	0.18	0.00 (−0.02, 0.02)	−0.04	0.68
	LDL (mmol/l)	138	**0.26**	**0.002**	0.04	0.67	**0.15 (0.03, 0.26)**	**0.27**	**0.011**	−0.01 (−0.04, 0.02)	−0.05	0.58
	HDL (mmol/l)	138	−0.12	0.17	0.08	0.33	−0.03 (−0.07, 0.02)	−0.12	0.27	0.01 (−0.01, 0.02)	0.11	0.25
Inflammatory marker	Uric acid (μmol/l)	140	**0.37**	**<0.0001**	−0.08	0.33	**27.2 (12.9, 41.55)**	**0.36**	**<0.0001**	−3.6 (−7.3, 0.1)	−0.17	0.05
Atherosclerosis marker	CIMT (mm)	128	**0.19**	**0.036**	0.02	0.84	0.009 (−0.012, 0.029)	0.09	0.40	0.000 (−0.005, 0.006)	0.02	0.86

*Data are Pearson's correlation coefficients (r) and respective p-values; and β coefficients (95% confidence intervals), standardized coefficients (Std β), and respective p-values. Associations that are statistically significant (p <0.05) are shown in bold*.

*ALT, alanine transaminase; AST, aspartate transaminase; CI, confidence interval; CIMT, carotid intima-media thickness; HbA1c, glycated hemoglobin; HDL, high-density lipoprotein cholesterol; LDL, low-density lipoprotein cholesterol*.

* *Models included A/G, TBF%, and age as independent variables; where the outcome was blood pressure, models adjusted for height instead of age*.

† *These parameters were log-transformed to approximate a normal distribution, in which case the percentage change in the outcome response was obtained using the formula exp(β) (Supplementary Methods)*.

‡ *β coefficients are shown in association with a change of 0.1 in the A/G*.

Except for the association with CIMT that was no longer significant, all other associations were corroborated by multiple regression models (Table 5). Every 0.1 increase in A/G was associated with a 16% reduction in insulin sensitivity (95% CI −24%, −6%; *p* = 0.002), as well as with increases of 3.44 μIU/ml in fasting insulin (*p* = 0.004), 31% in ALT (95% CI 16%, 46%; *p* < 0.0001), 17% in AST (95% CI 7%, 27%; *p* < 0.0001), 0.20 mmol/l in total cholesterol (*p* = 0.016), 0.15 mmol/l in LDL (*p* = 0.011), and 27 μmol/l in uric acid (*p* < 0.0001; Table 5).

### Adverse Cardiometabolic Outcomes

In pre-pubertal boys, every 0.1 increase in A/G was associated with odds of dyslipidaemia and NAFLD that were 1.6 and 2.6 times greater, respectively (Table 6). Among pubertal boys, the same increase in A/G was associated with increased odds of impaired glucose tolerance (aOR 1.64), abnormal glycaemia (aOR 1.51), and NAFLD (aOR 1.69) (Table 6). Among pubertal girls, the 0.1 unit increase in A/G was associated with greater odds of impaired glucose tolerance (aOR 1.81) (Table 6).

**Table 6:** The adjusted odds ratio (aOR) of adverse cardiometabolic outcomes in association with the android-to-gynoid-fat ratio (A/G) or total body fat percentage (TBF%) among children and adolescents with obesity in Hangzhou (China).

		Pre-pubertal boys^*^			**Pubertal boys**^**†**^			**Pubertal girls**^**‡**^		
		**Prevalence**	**aOR (95% CI)**	***p***	**Prevalence**	**aOR (95% CI)**	***p***	**Prevalence**	**aOR (95% CI)**	***p***
Impaired fasting glucose	A/G^**^	20.9%	0.89 (0.54, 1.46)	0.64	18.2%	1.12 (0.80, 1.57)	0.50	32.1%	1.20 (0.82, 1.76)	0.36
	Total body fat %		0.98 (0.83, 1.14)	0.76		1.03 (0.95, 1.11)	0.51		0.98 (0.88, 1.08)	0.65
Impaired glucose tolerance	A/G^**^	15.1%	1.42 (0.79, 2.55)	0.24	30.1%	**1.64 (1.07, 2.51)**	**0.022**	18.6%	**1.81 (1.10, 2.98)**	**0.021**
	Total body fat %		1.07 (0.89, 1.29)	0.49		1.03 (0.93, 1.14)	0.60		0.96 (0.84, 1.10)	0.57
Abnormal glycaemia	A/G ^**^	35.3%	1.06 (0.69, 1.62)	0.78	39.8%	**1.51 (1.08, 2.11)**	**0.016**	40.7%	1.42 (0.97, 2.09)	0.07
	Total body fat%		1.02 (0.89, 1.17)	0.78		1.00 (0.93, 1.08)	0.99		0.96 (0.87, 1.06)	0.41
Hypertension	A/G^**^	46.8%	1.03 (0.69, 1.54)	0.89	45.9%	0.88 (0.63, 1.21)	0.43	49.3%	1.24 (0.87, 1.77)	0.24
	Total body fat %		1.01 (0.89, 1.15)	0.83		1.02 (0.95, 1.10)	0.53		0.99 (0.90, 1.08)	0.83
Dyslipidaemia	A/G ^**^	48.2%	**1.62 (1.05, 2.49)**	**0.028**	48.3%	0.87 (0.64, 1.19)	0.39	47.9%	1.16 (0.81, 1.67)	0.41
	Total body fat %		0.94 (0.83, 1.07)	0.37		0.96 (0.89, 1.03)	0.29		0.95 (0.87, 1.05)	0.30
Metabolic syndrome	A/G^**^	n/a	n/a	n/a	45.5%	0.97 (0.71, 1.31)	0.83	44.3%	1.31 (0.90, 1.90)	0.15
	Total body fat%	n/a	n/a	n/a		0.97 (0.90, 1.05)	0.44		0.96 (0.88, 1.06)	0.46
NAFLD	A/G^**^	61.2%	**2.57 (1.56, 4.21)**	**0.0002**	59.1%	**1.69 (1.18, 2.41)**	**0.004**	49.3%	1.31 (0.88, 1.97)	0.18
	Total body fat %		1.14 (0.99, 1.31)	0.07		**1.15 (1.06, 1.26)**	**0.001**		1.02 (0.91, 1.12)	0.78
Hyperuricaemia	A/G^**^	8.6%	1.22 (0.59, 2.54)	0.59	24.4%	1.43 (0.98, 2.10)	0.06	12.1%	1.72 (0.97, 3.03)	0.06
	Total body fat%		1.02 (0.81, 1.28)	0.89		1.02 (0.94, 1.12)	0.62		0.93 (0.80, 1.10)	0.40

*The definitions of the adverse outcomes are provided in Table 1*.

*Statistically significant associations (p < 0.05) are shown in bold*.

*n/a, not applicable (current guidelines recommend that it is not appropriate to diagnose the metabolic syndrome in young children); CI, confidence interval; NAFLD, non-alcoholic fatty liver disease*.

* *Models included the A/G and TBF% as independent variables*.

† *Models included the A/G, TBF%, and testicular volume as independent variables; where the outcome was hypertension, models adjusted for height instead of testicular volume*.

‡ *Models included the A/G, TBF%, and age as independent variables; where the outcome was hypertension, models adjusted for height instead of age*.

** *aOR are shown in association with a change of 0.1 in the A/G*.

In contrast to A/G, TBF% was only an independent predictor of NAFLD in pubertal boys (aOR 1.15), and was not associated with the likelihood of any other cardiometabolic outcome assessed, irrespective of pubertal stage, or sex (Table 6).

### Summary of Findings

The observed independent associations between A/G or TBF% and cardiometabolic outcomes in our study are summarized in Table 7. In brief, increasing A/G was associated with lower insulin sensitivity and higher fasting insulin levels in all groups, as well as with greater odds of impaired glucose tolerance in pubertal boys and girls, and of abnormal glycaemia in pubertal boys (Table 7). In addition, increasing A/G was associated with high uric acid and ALT levels in all groups, higher total cholesterol and LDL levels in pubertal girls, and greater odds of NAFLD in pre-pubertal and pubertal boys (Table 7).

**Table 7:** Summary of the independent associations between the android-to-gynoid-fat ratio (A/G) or total body fat percentage (TBF%) and cardiometabolic outcomes among children and adolescents with obesity in Hangzhou (China).

		**Pre-pubertal boys**		**Pubertal boys**		**Pubertal girls**	
		**A/G**	**TBF%**	**A/G**	**TBF%**	**A/G**	**TBF%**
Glucose metabolism	Matsuda index	**↓**	–	**↓**	–	**↓**	–
	Fasting insulin (μIU/ml)	**↑**	–	**↑**	**↑**	**↑**	–
	HbA1c (mmol/mol)	**↑**	–	–	–	–	–
	Impaired glucose tolerance	–	–	**↑**	–	**↑**	–
	Abnormal glycaemia	–	–	**↑**	–	–	–
Liver function	ALT (U/l)	**↑**	–	**↑**	**↑**	**↑**	–
	AST (U/l)	–	–	–	–	**↑**	–
	NAFLD	**↑**	–	**↑**	**↑**	–	–
Lipid profile	Total cholesterol (mmol/l)	–	–	–	–	**↑**	–
	Triglycerides (mmol/l)	–	–	–	**↓**	–	–
	LDL (mmol/l)	–	–	–	–	**↑**	–
	Dyslipidaemia	**↑**	–	–	–	–	–
Inflammatory marker	Uric acid (μmol/l)	**↑**	**↑**	**↑**	–	**↑**	–
Atherosclerosis marker	CIMT (mm)	–	–	–	**↑**	–	–

*↑ indicates a statistically significant positive association and ↓ a negative association between A/G or TBF% and a given outcome, from either a multiple regression for continuous outcomes or a generalized linear model for categorical adverse outcomes*.

*ALT, alanine transaminase; AST, aspartate transaminase; CIMT, carotid intima-media thickness; HbA1c, glycated hemoglobin; cholesterol; LDL, low-density lipoprotein cholesterol; NAFLD, non-alcoholic fatty liver disease*.

*The definitions of dyslipidaemia, impaired glucose tolerance, and abnormal glycaemia are provided in Table 1*.

In contrast, in pre-pubertal boys, increasing TBF% was associated higher uric acid levels; in pubertal boys, TBF% was associated with higher fasting insulin and ALT levels, increased CIMT, and greater odds of NAFLD (Table 7). There were no observed associations between TBF% and any outcomes in pubertal girls when A/G was accounted for (Table 7).

## Discussion

In this cross-sectional study of 479 Chinese children and adolescents with obesity, A/G was a much stronger predictor of cardiometabolic health than TBF%, irrespective of pubertal status. Increasing A/G was associated with abnormalities in glucose homeostasis and liver function, as well as increased inflammation (i.e., uric acid levels) irrespective of pubertal status or sex. A/G was also predictive of NAFLD among all boys, but not girls. In contrast, TBF% was a poor predictor of cardiometabolic health among pre-pubertal boys and pubertal girls; while it was predictive among pubertal boys, the association with glucose homeostasis was considerably weaker than those observed for A/G.

Our findings corroborate the observations from smaller pediatric studies. A cross-sectional study of 66 French children with obesity reported that A/G was more strongly correlated with insulin resistance than TBF% (23), which was also observed among 73 US children aged 7 to 13 years across the BMI range (27). Another US study on 421 adolescents reported that greater abdominal obesity was associated with increased risk of metabolic syndrome (24). Conversely, Hetherington-Rauth et al. showed that among 232 Hispanic girls aged 9 to 12 years, TBF% and regional adiposity were similarly associated with cardiometabolic risk factors (25), although the authors did not include A/G among the parameters assessed.

These results are in agreement with other clinical data and our understanding of the importance of visceral adiposity and metabolic risk. In particular, visceral fat is more metabolically active (46), with increased circulating portal levels of free fatty acids (47). These have been shown to be a major cause for reduced insulin sensitivity and hepatic insulin resistance (48, 49). To maintain euglycaemia, portal secretion of insulin increases, resulting in higher systemic insulin levels, and subsequent peipheral insulin resistance (38, 39). Visceral fat is also associated with increased conversion of cortisone to cortisol, and the resulting increased cortisol levels are also associated with reduced insulin sensitivity and increased cardiovascular risk (50). Thus, it is not surprising that studies in adults and children have shown the A/G (a marker for visceral adiposity) is strongly associated with diseases linked to the metabolic syndrome. For instance, in Chinese adults, A/G and TBF% were both associated with cardiometabolic risk (22, 51), but A/G appears to be more closely associated with risk among women (51). Subcutaneous fat is not as metabolically active (46), and while there are metabolic deleterious consequences to generalized obesity, this study highlights the relevance of visceral adiposity and the importance of measuring indices of visceral fat.

Assessing insulin sensitivity is difficult, with varying definitions, and the focus with respect to glucose homeostasis is usually hyperglycaemia. However, reduced insulin sensitivity and the associated compensatory hyperinsulinism are early findings in patients developing the metabolic syndrome, and are fundamental to the pathogenesis of these conditions. While the hyperinsulinaemic euglycaemic clamp is the gold standard technique to measure whole-body insulin sensitivity, it is a complex and time-consuming procedure requiring numerous frequent blood samples (52). Thus, it is of limited use outside research settings, with other surrogate methods adopted instead (52), particularly HOMA-IR that relies solely on fasting plasma insulin and glucose samples (53). In this study, we used the Matsuda index to measure insulin sensitivity, which, as previously mentioned, has high reproducibility (36) and is strongly correlated with the clamp (35). In Asian adults, a number of studies have shown the Matsuda index to have a stronger correlation with the insulin sensitivity measured by the clamp compared to HOMA-IR (54–57). Importantly, the Matsuda index can accurately assess insulin sensitivity in children and adolescents (58). In this context, our results were similar to the findings of a previous study showing that insulin sensitivity was negatively correlated with A/G in children and adolescents, but was not associated with TBF% (23). However, one study reported that both the Matsuda index and HOMA-IR derived from cross-sectional data were poorly correlated with longitudinal assessments of insulin sensitivity (59). Therefore, some caution is required when using our findings to speculate on the potential long-term associations between adipose tissue distribution and insulin sensitivity.

Puberty is characterized by marked elevation in sex steroids, which are associated with a clear reduction in insulin sensitivity in both boys and girls (60–62). In the early stages of puberty, there are limited differences in body composition and cardiometabolic parameters between boys and girls (61, 63), although insulin sensitivity seems to be lower in girls (62, 63). Further, in pubertal girls insulin sensitivity in particular, is also affected by the menstrual cycle (64). Thus, clinical assessments can be confounded by these issues. The insulin sensitivity data in this study showed the expected reduction in insulin sensitivity in both sexes following pubertal onset, and a similar increase in insulin secretion to maintain euglycaemia (Table 2). The remaining descriptive cardiometabolic data for our cohort also show that, while there were limited sex differences, there were marked differences observed between pre-pubertal and pubertal groups (Table 2). This is likely a result of the relatively young age of our cohort, as clear sex differences develop in the late stages of pubertal development (61, 63). In addition, while females who are having regular menses should have insulin sensitivity performed in the follicular phase of their cycle, this timing was not recorded for this study. However, the similar pubertal Matsuda index for males and females suggests that this did not impact substantively on the results. Girls also enter puberty ~2 years earlier than boys, resulting in worsening insulin sensitivity, being probably a major reason why girls develop type 2 diabetes earlier than boys (65). Nonetheless, the key limitation of our study was the small number of pre-pubertal girls, so that we were unable to examine the associations between TBF% and A/G and cardiometabolic parameters in this group, or how such associations could change during puberty.

We also observed a strong association between increasing A/G ratio and greater uric acid levels in all subgroups. Some studies have shown that, compared to total body fat, abdominal adipose tissue is more strongly correlated with uric acid (66, 67). Our findings are consistent with a study in US children and adolescents that also reported an association between abdominal obesity and uric acid levels (68). In a state of obesity, there is increased xanthine oxidoreductase expression by abdominal adipose tissue, which is an important enzyme that catalyses the last two steps of uric acid synthesis (69). There is increased activation of xanthine oxidoreductase in association with adipose tissue accumulation, which causes local hypoxia (70), increasing uric acid levels (69). Abdominal adipose tissue is also more likely to lead to insulin resistance and hyperinsulinemia, which can increase uric acid reabsorption from the renal tubules, further increasing uric acid levels (71). Further, *in vivo* and *in vitro* studies have also shown that high uric acid levels can modulate glucose and lipid metabolism in liver, adipose tissue, and muscles, by increasing oxidative stress and activating immune cells (72). Therefore, it is not surprising that we observed higher uric acid levels with increased abdominal adiposity, irrespective of sex or pubertal development.

Our study included ultrasound CIMT assessment, which is a reproducible and validated measure that is predictive of cardiovascular and cerebrovascular risks (38). There is some evidence that severe obesity is associated with stiffness and endothelial dysfunction of arterial wall in children (73). However, CIMT may not be a particularly useful parameter in children, and a review reported no associations between CIMT and TBF% or visceral fat in pre-pubertal children, but there was an observed association with adiposity in late adolescence (74). Similarly, in our study, CIMT was only correlated with TBF% among pubertal boys. McGill et al. discussed that atherosclerosis begins in childhood, starting from aortic “fatty streaks” (deposits of cholesterol and its esters) that over time progress to atherosclerotic lesions in adults (75). Therefore, the observed associations between increasing adiposity and greater CIMT are suggestive of an increased atherosclerotic process in many adolescents with obesity, which could potentially lead to greater cardiovascular morbidity and mortality in the long-term.

Of note, the prevalence of cardiometabolic abnormalities was high among children with obesity, consistent with previous studies in China (76, 77). As expected by the greater reduction in insulin sensitivity, higher insulin levels, and greater metabolic disturbance, the prevalence of CVS abnormalities was higher in adolescents in this study (78, 79). For all the complications, NAFLD was the only variable that correlated with both TBF% and A/G in boys. There was a good correlation between hyperuricemia and A/G in adolescent girls, while complications of glucose metabolism and abnormal lipid metabolism were not found to be significantly correlated with A/G and TBF%. We hypothesize that these results may reflect the diagnostic criteria of IDF. When adjusting for height there is a difference in the inter-ethnic A/G (80), and thus complications should be diagnosed according to ethnically-specific diagnostic criteria.

Apart from the under-representation of pre-pubertal girls and the lack of follicular phase data, additional limitations of our study included our population of children and adolescents with obesity brought to our clinic by their parents, rather than consisting of randomly selected participants. While our population displayed high rates of cardiometabolic abnormalities, our observations were consistent with previous studies in China (76, 77). Nonetheless, our data was cross-sectional, and information on the participants' dietary habits, physical activity levels (81), or detailed obesity history was not collected. This cross-sectional design also limits our ability to infer causal associations or temporality between abdominal adiposity and cardiometabolic health. In addition, there seems to be a strong genetic influence on adipose tissue distribution (82), and we cannot ascertain the extent to which genetic factors might have affected the associations reported in this study. Further, as all participants in this study were Han Chinese, the results cannot be readily extrapolated to other ethnicities, as there are reported ethnic differences in fat distribution (83).

In summary, our study showed that A/G was a much stronger independent predictor of cardiometabolic outcomes in Chinese children and adolescents with obesity in comparison to TBF%, particularly glucose metabolism in pubertal individuals. However, longitudinal studies would help clarify whether the observed associations will mirror outcomes later in adulthood.

## Data Availability Statement

The datasets presented in this article are not readily available because of the conditions of the ethics approval. The anonymized data on which this article was based could be made available to other investigators upon bona fide request, and following all the necessary approvals (including ethics) of the detailed study proposal and statistical analyses plan. Requests to access the datasets should be directed to Prof. Junfen Fu, fjf68@zju.edu.cn.

## Ethics Statement

The studies involving human participants were reviewed and approved by the Medical Ethics Committee of the Children's Hospital of Zhejiang University School of Medicine the Children's Hospital, Zhejiang University School of Medicine, National Clinical Research Center for Child Health. Written informed consent to participate in this study was provided by the participants' legal guardian/next of kin.

## Author Contributions

JF was responsible for funding acquisition. JF, JD, BJ, and WC contributed to study design. HL, JY, GD, KH, WW, XC, LZ, JW, XL, YD, XX, XZ, MZ, GL, and JF carried out the clinical assessments. BJ, HL, JY, and JD were responsible for data curation. JD and BJ analyzed the data. BJ, JD, and PH wrote the manuscript, which was critically reviewed by all other authors.

## Conflict of Interest

The authors declare that the research was conducted in the absence of any commercial or financial relationships that could be construed as a potential conflict of interest.
