# Supplementary material for: Abdominal Adiposity and Total Body Fat as Predictors of Cardiometabolic Health in Children and Adolescents With Obesity
Source: Front Endocrinol (Lausanne). 2020 Sep 4;11:579. doi: 10.3389/fendo.2020.00579 (PMC7498567; doi:10.3389/fendo.2020.00579)
Supplement: Supplementary file 1 [file Data_Sheet_1.pdf]

Supplementary File

Abdominal adiposity and total body fat as predictors of cardiometabolic health in children and adolescents with obesity

Binghan Jin[1], Hu Lin[1], Jinna Yuan[1], Guanping Dong[1], Ke Huang[1], Wei Wu[1], Xuefeng Chen[1], Li Zhang[1], Jinling Wang[1], Xinyi Liang[1], Yangli Dai[1], Xiaoqin Xu[1], Xuelian Zhou[1], Mingqiang Zhu[1], Guohua Li[1], Wayne S Cutfield[1,2,3], Paul L Hofman[2], José G B Derraik[1,2,3,4*], Junfen Fu[1,*]

[1] Endocrinology Department, Children's Hospital of Zhejiang University School of Medicine, National Clinical Research Center for Child Health, Hangzhou, China

[2] Liggins Institute, University of Auckland, Auckland, New Zealand.

[3] A Better Start – National Science Challenge, University of Auckland, New Zealand

[4] Department of Women's and Children's Health, Uppsala University, Uppsala, Sweden

*Authors for correspondence:

Dr José Derraik – j.derraik@auckland.ac.nz

Prof Junfen Fu – fjf68@zju.edu.cn; fjf68@qq.com

Supplementary Methods

Calculation and interpretation of β-coefficients for log-transformed outcomes

The table below displays the output from the linear regression model for pre-pubertal boys in Hangzhou, where the log-transformed Matsuda index was the outcome, and the android-to-gynoid-fat ratio (A/G) and total body fat percentage (TBF%) were the predictors.

					**β: 95% CI**	
	**Unstd β**	**SEM**	**Std β**	***p***	**Lower**	**Upper**
**(Constant)**	5.81	1.04		<0.0001	3.76	7.85
**A/G ***	-0.34	0.06	-0.46	<0.0001	-0.45	-0.22
**TBF%**	-0.03	0.02	-0.11	0.14	-0.06	0.01

*β coefficients are shown in association with a change of 0.1 in A/G.

As the outcome was log-transformed, the unstandardised β coefficient and the respective 95% CI have to be back-transformed, so that the back-transformed parameters for A/G are:

β coefficient = exp (-0.34) = 0.71
Lower boundary = exp (-0.45) = 0.64
Upper boundary = exp (-0.22) = 0.80

Thus, for every 0.1 increase in A/G ratio, the model estimates that insulin sensitivity would increase by 0.71 times, i.e. 29% lower (95% CI 20%, 36%).

Supplementary Table 1

Pearson's correlation coefficients (*r*) and respective p-values for the associations between the android-to-gynoid-fat ratio and total body fat percentage among children and adolescents in Hangzhou (China) according to sex and pubertal status.

		n	*r*	*p*
**Overall**		479	-0.01	0.78
**Pubertal**	**All**	323	0.10	0.20
	**Boys**	183	0.45	0.07
	**Girls**	140	-0.01	0.91
**Pre-pubertal**	**All**	156	-0.01	0.87
	**Boys**	139	0.29	<0.001
	**Girls**	17	-0.13	0.07

Supplementary Figure 1

The distribution of body mass index standard deviation scores (BMI SDS) and total body fat percentage among children and adolescents with obesity in Hangzhou (China), according to sex and pubertal status.

Pre-pubertal boys (n=139); pubertal boys (n=183); and pubertal girls (n=140). Horizontal bars represent the medians and the interquartile ranges.

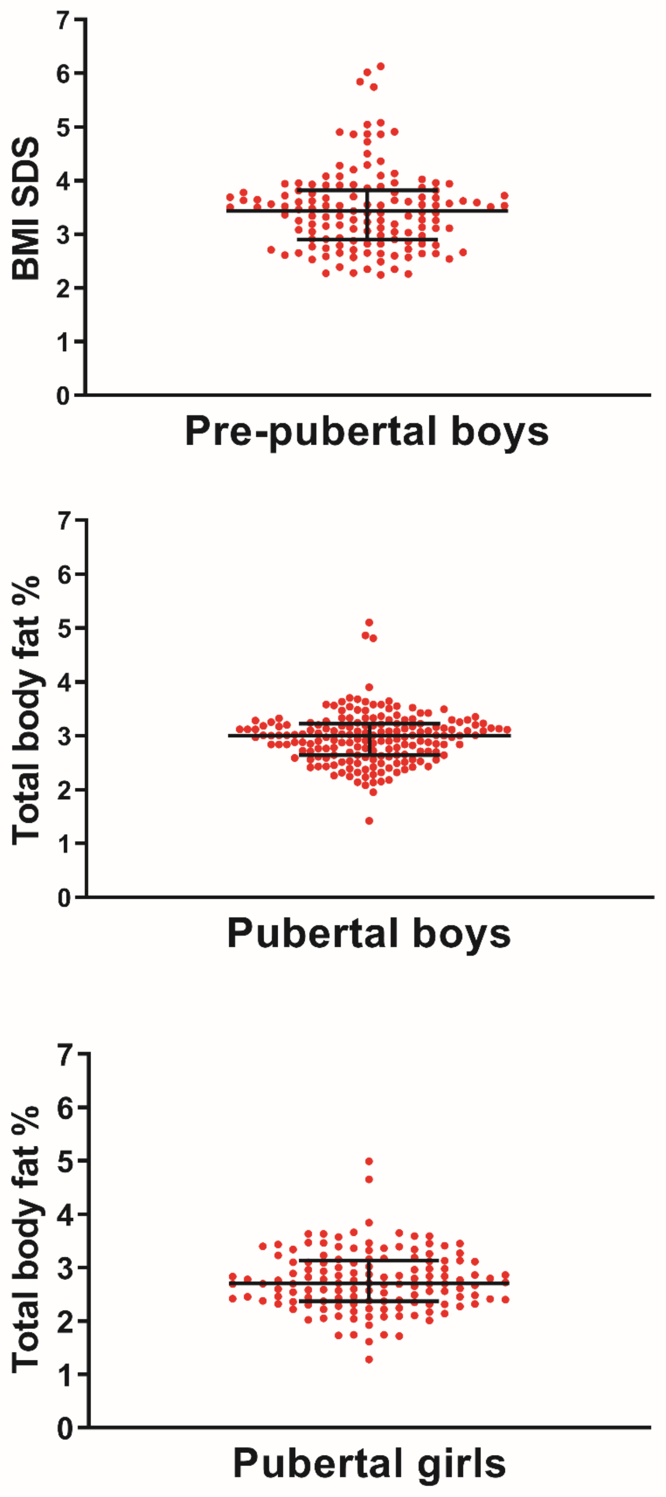

Jin B, Lin H, Yuan J, Dong G, Huang K, Wu W, Chen X, Zhang L, Wang J, Liang X, Dai Y, Xu X, Zhou X, Zhu M, Li G, Cutfield WS, Hofman PL, Derraik JGB, Fu J. Abdominal adiposity and total body fat as predictors of cardiometabolic health in children and adolescents with obesity. Frontiers in Endocrinology.

Supplementary Figure 2

The distribution of measured android-to-gynoid-fat ratio (A/G; blue) and total body fat percentage (red) among children and adolescents with obesity in Hangzhou (China), according to sex and pubertal status.

Pre-pubertal boys (n=139); pubertal boys (n=183); and pubertal girls (n=140). Horizontal bars represent the medians and the interquartile ranges.

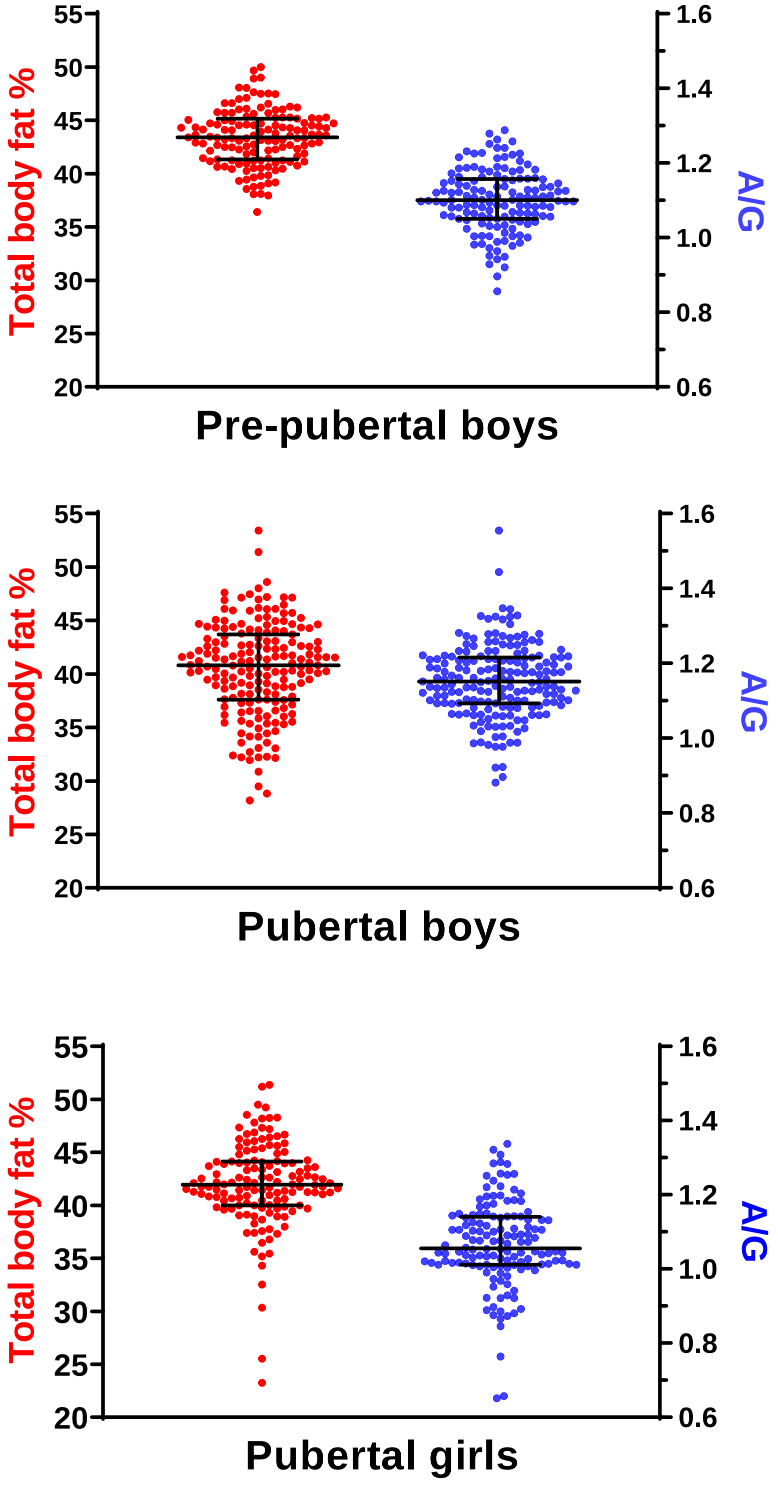

Jin B, Lin H, Yuan J, Dong G, Huang K, Wu W, Chen X, Zhang L, Wang J, Liang X, Dai Y, Xu X, Zhou X, Zhu M, Li G, Cutfield WS, Hofman PL, Derraik JGB, Fu J. Abdominal adiposity and total body fat as predictors of cardiometabolic health in children and adolescents with obesity. Frontiers in Endocrinology.

Supplementary Figure 3

Linear associations between both the android-to-gynoid-fat ratio and total body fat percentage and insulin sensitivity assessed by the Matsuda index among children and adolescents with obesity in Hangzhou (China).

A) Pre-pubertal boys (n=139); B) Pubertal boys (n=183); and C) Pubertal girls (n=140).
Matsuda index data were log-transformed to approximate normality.

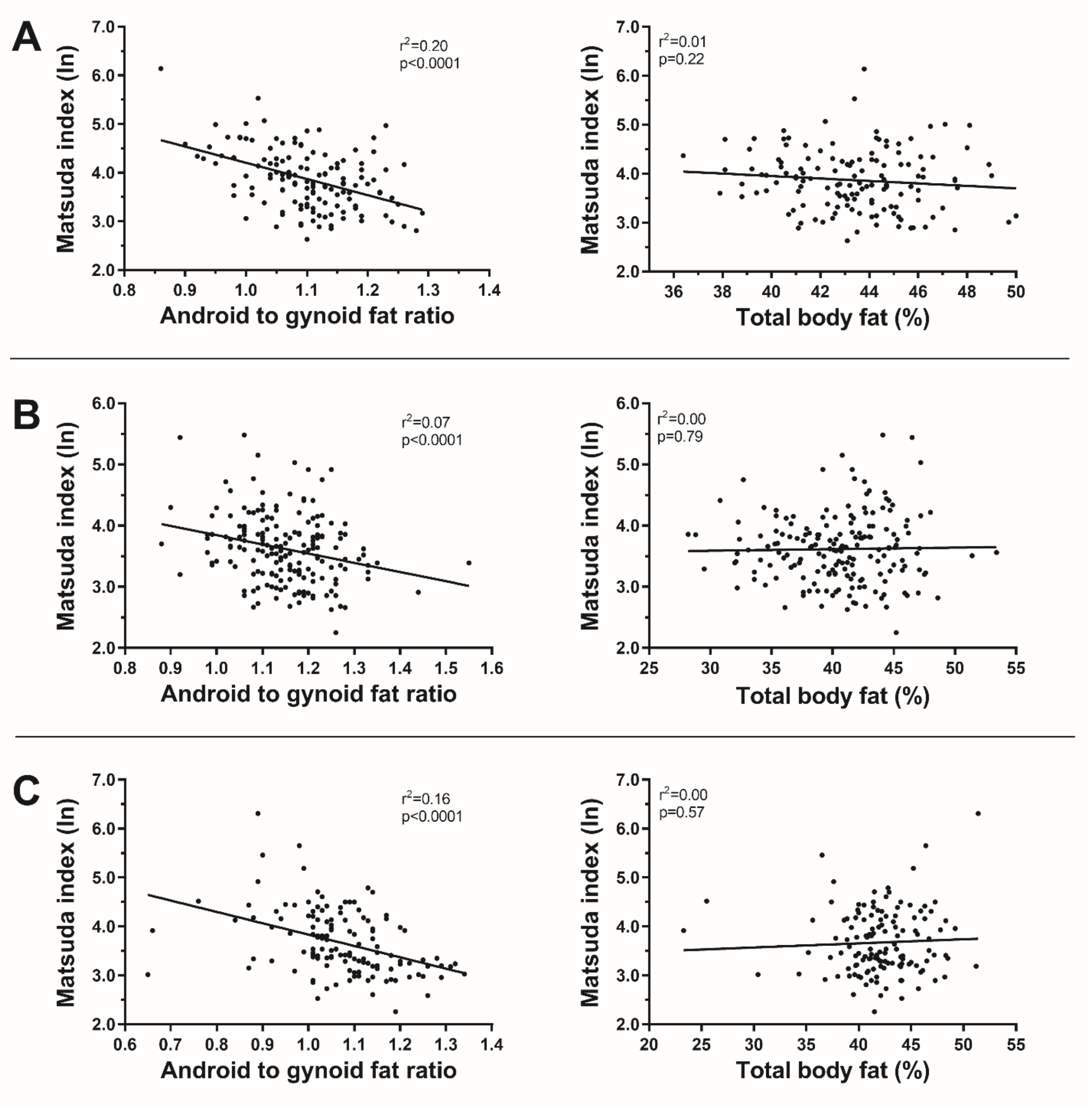

Jin B, Lin H, Yuan J, Dong G, Huang K, Wu W, Chen X, Zhang L, Wang J, Liang X, Dai Y, Xu X, Zhou X, Zhu M, Li G, Cutfield WS, Hofman PL, Derraik JGB, Fu J. Abdominal adiposity and total body fat as predictors of cardiometabolic health in children and adolescents with obesity. Frontiers in Endocrinology.
